# A Time–Frequency Energy Squeeze Method Based on Estimating the Chip Rate of DSSS Signals

**DOI:** 10.3390/s25030596

**Published:** 2025-01-21

**Authors:** Hang Zhao, Feng-Yuan Sun, Yuan Cai, Wei-Kun Liu

**Affiliations:** 1 Guangxi Key Laboratory of Precision Navigation Technology and Application, School of Information and Communication, Guilin University of Electronic Technology, Guilin 541004, China; z2753986011@163.com (H.Z.); 13557777241@163.com (W.-K.L.); 2 School of Information and Communication, Guilin University of Electronic Technology, Guilin 541004, China; ssccyyss@aliyun.com

**Keywords:** DSSS signals, wavelet function, energy squeeze, chip rate estimation

## Abstract

Chip rate estimation for direct-sequence spread spectrum (DSSS) signals plays a crucial role in signal detection and interference identification in non-cooperative wireless communication systems. However, accurate chip rate estimation is difficult in low signal-to-noise ratio (SNR) environments due to the influence of noise and channel fading. To address this problem, an improved chip rate estimation method, based on a time-reassigned multisynchrosqueezing transform energy squeeze, for DSSS signals is proposed for low SNR conditions. In this method, we explain how to choose an appropriate wavelet function and concentrate the wavelet coefficients on the energy ridge by reordering time details. This enhances the time resolution of the signal energy, reduces noise interference, and improves the accuracy of chip rate estimation for DSSS signals. To validate the proposed method, we compared the effects of different information code lengths, spread spectrum code lengths, spread spectrum code sequences, and modulation schemes on chip rate estimation performance with traditional methods. Additionally, we applied the method to real DSSS signal data to demonstrate its feasibility. Simulation examples and measurements demonstrate the reliability of the proposed method in low SNR environments.

## 1. Introduction

The direct-sequence spread spectrum (DSSS) technology has been widely used in military and civil communication due to its anti-jamming capability, especially in non-cooperative wireless communication systems. In these applications, chip rate estimation is an important post-processing signal, which is often used for signal detection and interference identification. However, with the need for greater communication requirements in complex environments, the accuracy of chip rate estimation is facing great challenges due to the intricacies of communication channels and strong interference. These challenges arise from factors such as channel transmission noise interference, which can degrade signal quality and make accurate chip rate estimation difficult, particularly in low signal-to-noise ratio (SNR) conditions.

The classical methods for DSSS signal chip rate estimation can be classified into three categories: time domain, frequency domain, and time-frequency (TF) domain. These methods are further refined with delay multiplied auto-correlation, envelope spectrum, cyclic spectrum, matrix decomposition, and wavelet analysis [1,2,3,4,5]. For the time domain method, delay multiplied auto-correlation utilizes the inherent periodicity and auto-correlation of DSSS signals to effectively estimate the chip rate [1]. However, the performance of this method is highly sensitive to the selection of an appropriate delay length, and it is particularly vulnerable to noise interference in low SNR environments. In the case of frequency domain methods, the cyclic spectrum method makes use of the cyclic spectral properties of both the signal and the noise, demonstrating strong robustness against noise and interference [2]. Nevertheless, this method is computationally intensive and suffers from poor real-time performance. The envelope spectrum method effectively captures the transient characteristics of signals, enabling accurate chip rate estimation. Nonetheless, due to its limited high-frequency resolution, envelope spectrum analysis struggles to effectively capture transient changes in signals when in low SNR surroundings [3]. Time–frequency domain methods combine the advantages of both time and frequency domains, allowing for a more comprehensive analysis of signal characteristics. The matrix decomposition technique effectively analyzes the correlation features of signals but may be susceptible to numerical instability, leading to inaccurate results [4]. Wavelet analysis, as a typical representation of time–frequency analysis, has demonstrated superior performance in symbol rate estimation compared to other techniques, and it has the advantage of not requiring prior information about the signal [5,6]. The major reason for this superior performance is that a TF window can be adapted to different frequencies. In the vicinity of the signal jump point, the wavelet transform shows localized characteristics, which are usually manifested as a sudden increase or decrease in the coefficients. Combined with the flexibility of the TF window, the jump information in the signal can be effectively extracted by detecting this change. Based on this advantage, it can be used to identify the location of phase transitions in a signal and estimate the chip rate of the signal by analyzing the wavelet transform coefficients.

Regarding the applications of wavelet analysis in symbol rate estimation, many researchers have already conducted relevant studies. In early studies, the Haar wavelet-based method was proposed for blind symbol rate estimation [7], where the accuracy of estimation was improved by combining coefficients from multiple scales, especially for M-ary phase-shift keying (MPSK) signals. However, the Haar wavelet-based method has limitations when applied to other signal types and performs poorly at low signal-to-noise ratios (SNRs) [8,9]. Considering that the DSSS signal is also MPSK signal in essence, the Haar wavelet power spectrum method was proposed for a blind estimation of the DSSS signal chip rate [10]. This method offers superior performance and lower computational complexity compared to the cyclic spectrum method. Additionally, the use of a Morlet wavelet can also improve estimation accuracy, but it increases the computational complexity due to prior estimation of the optimal scaling factor [11]. Traditional wavelet transform techniques suffer from poor energy concentration, which significantly limits their accuracy in noisy environments. In general, methods based on TF analysis are more sensitive than other methods in identifying phase transition. But the dispersion of the signal energy across the TF plane is induced usually in traditional TF transformations, which results in the fuzzification or dispersion of main signal features. This dispersion reduces the resolution and accuracy in capturing the signal features, making it challenging to analyze the primary characteristics of the signal. To solve the problem of energy dispersion in the TF domain, the TF reassignment method was proposed as a post-processing method [12]. Subsequently, methods based on frequency rearrangement and time rearrangement were proposed to improve the energy concentration in TF representations while preserving signal reconstruction capability [13,14]. These methods effectively isolate the noise around ridge curves, thereby enhancing robustness against noise interference.

In this letter, we propose a method based on TF analysis energy compression to reduce the influence of noise on estimating the chip rate of DSSS signals. Firstly, an appropriate wavelet basis function is selected to rearrange the wavelet coefficients, concentrating the signal energy onto the energy ridge to improve the signal’s resolution. Secondly, the amplitude spectrum of the rearranged wavelet coefficients is analyzed to estimate the chip rate. Finally, to validate our approach, we compared it with traditional wavelet analysis methods using both synthetic signals generated by simulation software and real signals captured with an RTL-SDR device. This comparison demonstrates the method’s robustness and enhanced its applicability.

This study is organized as follows: Section 2 reviews the signal model; Section 3 presents the proposed methods; Section 4 and Section 5 analyze the theoretical and practical simulation results, respectively; and Section 6 summarizes this paper.

## 2. Signal Model

The DSSS signal is modeled as follows:

(1) $$S_{DSSS}\left(t\right)=d\left(t\right)c\left(t\right)=\sum_{n=0}^{\infty }C_{n}g_{c}\left(t-nT_{c}\right),$$

where d(t) and c(t) are the message signal and the pseudo-random sequence, respectively; d(t) represents binary digital information; c(t) is generated by a pseudo-random sequence generator; the product of $d_{n}$ and $c_{n}$ is denoted as $C_{n}$; and the length of the information symbol sequence is $T_{d}$, while $T_{c}$ represents the length of the pseudo-random sequence. Generally, the chip rate of the spread spectrum signal $S_{DSSS}\left(t\right)$ is much higher than that of the message signal d(t). Consequently, the spread spectrum period remains equal to the period $T_{c}$ of the pseudo-random sequence, and the chip rate of the spread spectrum sequence is equal to $R_{c}=\frac{1}{T_{c}}$.

To analyze the received signal, we assumed that the received DSSS signal was modulated with a binary phase-shift keying (BPSK). In this case, the signal can be represented as follows:

(2) $$\begin{array}{c} y(t)=A\sum_{n}C_{n}g_{c}\left(t-nT_{c}\right)e^{j\varphi_{s}}e^{j(\omega_{c}t+\varphi_{0})}+\eta \left(t\right), \end{array}$$

where the received signal y(t) is characterized by a series of rectangular pulses, denoted as $g_{c}\left(t\right)$, each with a pulse width of $T_{c}$; and parameters *A*, $\varphi_{s}$, $\omega_{c}$, and $\varphi_{0}$ represent the amplitude, the phase of the *i*-th symbol, angular frequency, and initial phase of the carrier, respectively. Furthermore, η(t) represents additive white Gaussian noise (AWGN), which is used to model the environmental interference that is typically encountered in non-cooperative communication systems.

## 3. Propose Method

The sensitivity of the wavelet transform to irregularities in signals makes it suitable for a blind estimation of the chip rate. Specifically, it captures transient and localized features, which is critical for accurate estimation. However, in TF analysis, the distribution of the signal energy across a wide range of time and frequency, due to the diverse shapes and multi-scale characteristics of wavelet basis functions. can obscure signal features, thus reducing parameter detection and estimation accuracy. To overcome this limitation, this study proposes a novel hybrid approach that integrates the wavelet transform with the time-reassigned multisynchronous transform. The wavelet transform captures signal irregularities and transient features, while the time-reassignment mechanism concentrates TF energy near the instantaneous frequency. This complementary strategy not only mitigates the effects of noise and distortion, but also enhances the robustness and accuracy of analysis under low SNR conditions.

The continuous wavelet transform (CWT) of the received signal y(t) is given as

(3) $$\begin{array}{c} W(a,b)=\frac{1}{\sqrt{a}}\int_{-\infty }^{\infty }y\left(t\right)\psi^{*}\left(\frac{t-b}{a}\right)dt, \end{array}$$

where *a* and *b* denote the scaling and translation factors, respectively, while $\psi^{*}\left(t\right)$ represents the conjugate of the wavelet function. The choice of wavelet basis plays a crucial role in determining the accuracy and efficiency of TF analysis. Gaussian-shaped window functions are particularly effective for transient signal analysis as they achieve superior energy concentration in the TF domain [14]. Accordingly, this study compared the performance of the Mexican Hat, Meyer, and Morlet wavelet basis in terms of chip rate for DSSS signals under identical simulation conditions. As shown in Table 1, the Morlet wavelet basis had the shortest computation time, demonstrating its computational efficiency. Consequently, this study utilized the Morlet wavelet basis for signal feature extraction, which is defined as $\psi \left(t\right)=Ag\left(t\right)e^{j\omega_{0}t}=Ae^{-\frac{t^{2}}{2}}e^{j\omega_{0}t}$. This wavelet structure combines computational efficiency with effective energy localization, making it an optimal choice for DSSS signal analysis within the proposed framework. Unlike traditional approaches, the proposed framework uniquely integrates the Morlet wavelet with a time-reassignment mechanism, ensuring superior energy concentration in the TF domain and robust chip rate estimation, even under challenging low SNR conditions.

The time domain expression of the continuous wavelet transform was derived by incorporating the Morlet wavelet basis function into Equation (3), as shown in Equation (4).

(4) $$\begin{array}{c} W(a,b)=\frac{1}{a}\int_{-\infty }^{+\infty }y\left(t\right){\left(g\left(\frac{t-b}{a}\right)e^{i\omega_{0}\frac{t-b}{a}}\right)}^{*}dt. \end{array}$$

To analyze the TF characteristics of the signal in depth, Parseval’s theorem can be used to derive the expression of the CWT in the frequency domain and the corresponding spectrum, namely Equations (5) and (6).

(5) $$\begin{array}{c} W_{s}\left(a,b\right)=\frac{a}{\sqrt{2\pi }}\int_{-\infty }^{\infty }Y\left(\xi \right)e^{-i\xi t+\frac{j\omega^{2}}{2}}d\xi , \end{array}$$

(6) $$\begin{array}{c} P(a,b)=W_{s}\left(a,b\right)W_{s}^{*}\left(a,b\right)=\int \int E_{a,b}\left(t,\xi \right)dtd\xi . \end{array}$$

### 3.1. Energy Compression Based on Time-Reassigned Multisynchronous Transform

The goal of the time operator and energy compression is to concentrate the dispersed signal energy along the time–frequency ridge, which improves the resolution for more accurate chip rate estimation. The concept of the energy distribution function can be used to describe the phenomenon of signal energy dispersion caused by wavelet transform. The function defines the center of gravity of the signal’s energy distribution around the TF point as the time and frequency operators. The modulus peaks of wavelet transform coefficients are observed at integer multiples of the symbol period. Thus, increasing the time resolution of the wavelet transform allows for a more accurate capture of the signal’s transient characteristics. Based on this, the time operator is selected to rearrange the wavelet TF coefficients. In this case, the time operator, also known as the group delay (GD) operator, represents the center of gravity of the actual position of the signal at each frequency. It is defined as

(7) $$\begin{array}{c} \hat{t}\left(a,b\right)=\frac{\;\int \int \;tE_{a,b}\left(t,\xi \right)dtd\xi }{\;\int \int \;E_{a,b}\left(t,\xi \right)dtd\xi }=b+\frac{aW_{s}^{tg}\left(a,b\right)}{W_{s}\left(a,b\right)}. \end{array}$$

To achieve the energy compression effect, we focused solely on the post-processing strategy along the time direction. The diffuse energy was compressed over energy ridge by calculating the GD operator, which rearranges the wavelet transform TF coefficients along the direction of the time axis to the position indicated by the GD operator [14], as shown in Equation (8).

(8) $$S\left(a,u\right)=\int W_{s}\left(a,b\right)\delta \left(u-\hat{t}\left(a,b\right)\right)db.$$

From Equation (1), the analytical signal in the frequency domain can be expressed as $S\left(\omega \right)=AS_{DSSS}\left(\omega \right)e^{j\psi (\omega )}$. The energy ridge is the GD operator given by $t\left(\omega \right)=-\psi^{\prime }\left(\omega \right)=-\frac{d\psi (\omega )}{d\omega }$ [15]. The smaller the difference between the actual $\hat{t}\left(a,b\right)$ and the theoretical $-\psi^{\prime }\left(\omega \right)$, the higher the concentration of signal energy. To minimize the error between $\hat{t}\left(a,b\right)$ and $-\psi^{\prime }\left(\omega \right)$, an *N*-th order iteration is applied to the time operator. Through continuous iterations, the time operator can be dynamically adjusted. The time operator after consecutive iterations is therefore given by the following:

(9) $$t^{\left[N\right]}\left(a,b\right)=-\varphi^{\prime }\left(\omega \right)+{\left[\frac{\varphi^{\prime \prime }{\left(\omega \right)}^{2}}{\varphi^{\prime \prime }{\left(\omega \right)}^{2}+{\left(a^{2}\sigma \right)}^{2}}\right]}^{N}\left(\varphi^{\prime }\left(\omega \right)+b\right).$$

When $\lim_{N\to \infty }{\hat{t}}^{\left[N\right]}\left(a,b\right)=-\varphi^{\prime }\left(\omega \right)$, this indicates that the signal energy concentration reaches the highest at present, and the TF characteristic of the DSSS signal becomes clearer and easier to interpret, which facilitates subsequent signal processing and analysis. Therefore, by substituting Equation (9) into Equation (8), the new CWT coefficient is expressed.

(10) $$\lim_{N\to \infty }S^{\left[N\right]}\left(a,u\right)=\int_{-\infty }^{+\infty }W_{s}\left(a,b\right)\delta \left(u+\varphi^{\prime }\left(\omega \right)\right)db.$$

### 3.2. Chip Rate Estimation

Due to the energy squeeze applied by the time-reassigned multisynchrosqueezing transform, the signal’s constant amplitude is significantly greater than the amplitude of abrupt changes during chip transitions. As a result, the amplitude of the signal can be expressed as follows:

(11) $$y\left(t\right)=\sum_{i}A_{i}\delta \left(t-iT_{s}\right).$$

Here, $T_{s}$ denotes the symbol width, $A_{i}$ represents the amplitude at the symbol jump point, and δ(t) is the impulse function. By applying the Fourier transform to Equation (11), the phase change during symbol hopping is converted into frequency change during chip hopping, as shown in Equation (12). This conversion allows for a more detailed analysis of the signal’s frequency characteristics.

(12) $$Y\left(\omega \right)=\frac{2\pi }{T_{s}}\sum_{n}B_{n}\delta \left(\omega -\frac{2n\pi }{T_{s}}\right),$$

where $B_{n}$ is the magnitude of the discrete spectrum at the *n*-th frequency component. The chip rate $1/T_{s}$ is estimated by identifying the peaks in the discrete spectrum. These discrete spectral peaks correspond to the chip rate and its multiples, respectively. The primary peak, located at $\omega =2\pi /T_{s}$, corresponds to the chip rate itself and reflects the basic periodicity of the signal. Higher-order harmonics appear at integer multiples of the fundamental frequency, specifically at $\omega =2n\pi /T_{s}$ (where *n*=2,3,…). These higher-order harmonics arise from rapid amplitude changes during chip transitions, which further emphasize the signal’s periodic structure. By identifying these discrete peaks in the spectrum, we can accurately estimate the chip rate of DSSS signals.

## 4. Simulation Result and Analysis

This section presents the simulation results of the proposed algorithm and evaluates its efficacy by comparing it with the algorithms in [10,11], which use the Morlet and Haar wavelet bases for TF analysis, respectively. The performance was evaluated using 100 Monte Carlo simulation runs, with SNR values ranging from −20 dB to 0 dB. For the simulations, an m-sequence with a spreading factor of 100 and a length of 127 was used to spread 15 data bits, which were then modulated using binary phase-shift keying and transmitted through an AWGN channel. The number of iterations, N, was set to 12, with a simulation error tolerance of 0.01%.

**Figure 1:**
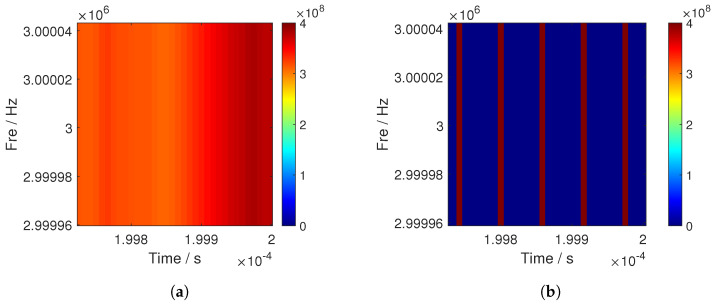
Time−frequency characterization plot of a DSSS-modulated signal. (**a**) Time−frequency plot before rearrangement. (**b**) Time−frequency plot after-rearrangement.

Figure 1a illustrates the TF analysis diagram obtained through CWT. In this case, the energy of the transient components is distributed, leading to poor signal resolution. This makes it challenging to accurately estimate the correct chip rate in strong noise. Figure 1b shows the TF analysis obtained after the time-rearrangement of the wavelet coefficients. It is evident that the energy of the transient components becomes more concentrated and the surrounding noise is significantly reduced, which offers advantages for chip rate estimation.

**Figure 2:**
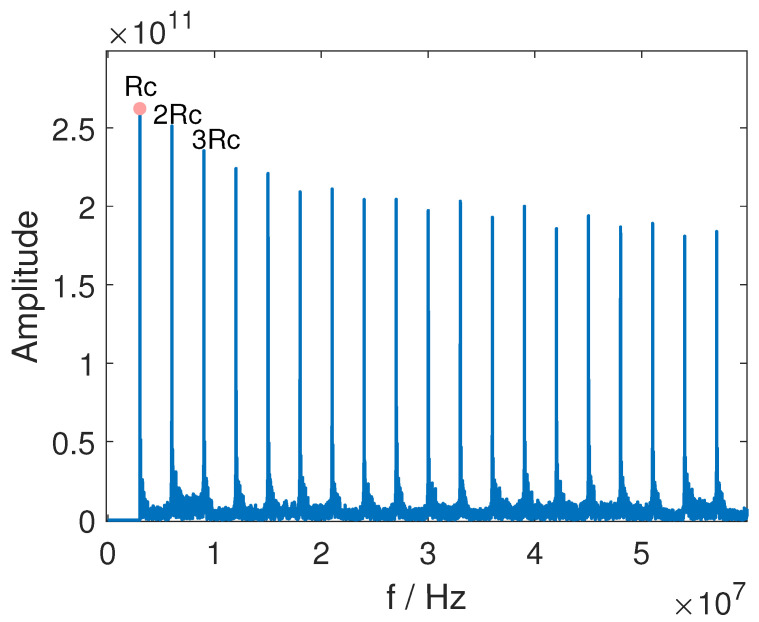
The power spectrum of DSSS signals from Equation (12).

Figure 2 displays the magnitude spectrum of Equation (12), where discrete spectral lines are observed at integer multiples of the chip rate. These lines are clearly visible and identifiable, making it easy to determine the chip rate by locating the position of the initial spectral line.

**Figure 3:**
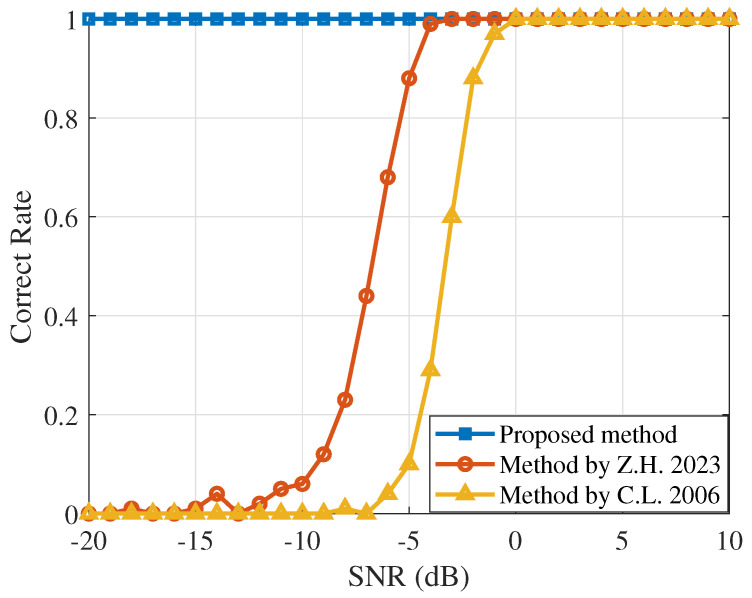
Performance of three different algorithms at different SNRs. The square line, circle line, and upper triangle line represent the outcomes of three different methods: the algorithm proposed in this paper, the algorithm estimated using the Morlet wavelet transform power spectrum method [11], and the algorithm estimated using the Haar wavelet transform power spectrum method [10], respectively.

Figure 3 shows the accuracy of chip rate estimation for different SNRs. The proposed method has better estimation performance than the conventional method.

**Figure 4:**
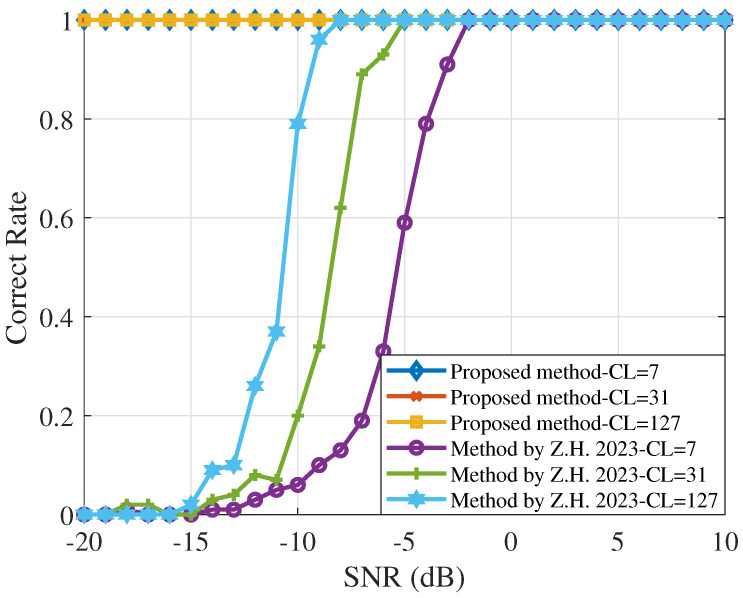
Performance curve of chip rate estimation for different information code lengths, where CL is the length of the selected information code. Diamonds, double vertical lines, and squares represent the proposed algorithm. Hollow circles, single vertical lines, and hexagons represent the Morlet wavelet transform power spectrum method [11].

**Figure 5:**
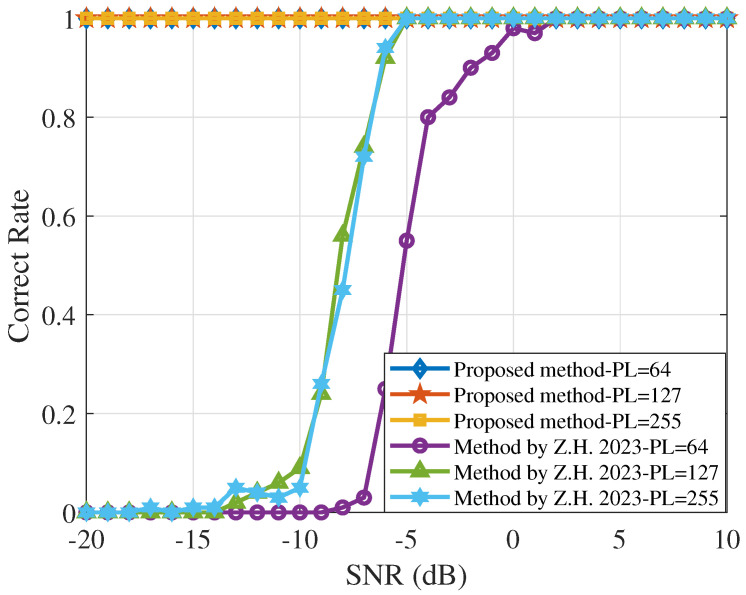
Performance curve of the chip rate estimation for different spread code lengths, where PL is the length of the selected spread code. Diamonds, pentagram, and squares represent the proposed algorithm, while hollow circles, upward-pointing triangles, and hexagons represent the Morlet wavelet transform power spectrum method [11].

**Figure 6:**
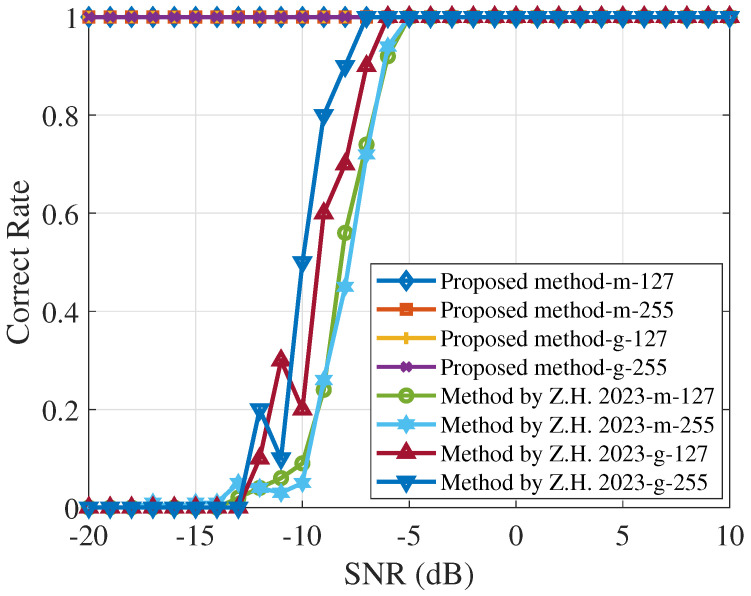
The performance curve of the chip rate estimation when using the m-sequence and Gold sequence of the same length is shown, where the proposed algorithm and the Morlet wavelet transform power spectrum algorithm [11] are compared.

Similarly, Figure 4, Figure 5 and Figure 6 analyze the comparison of the performance of DSSS signals using the proposed method and the Morlet wavelet transform power spectrum method, considering various information code lengths, spreading code lengths, and spreading sequences at different SNRs. Figure 4 compares the results of estimating the DSSS signal chip rate for information code lengths of 7, 31, and 127. A longer message code indicates more useful information. As the code length increases, the signal structure becomes more distinguishable from noise, improving the reliability of chip rate peak detection. It is observed that the accuracy of the chip rate estimation improves as the number of information codes increases. The proposed algorithm significantly outperforms the Morlet wavelet transform power spectrum method at SNRs below −9 dB.

Figure 5 compares the chip rate estimation results for spread spectrum code lengths of 64, 127, and 255 across different SNR levels. The longer the spreading code, the wider the spectral distribution of the DSSS signal, which improves its anti-interference capability. The Morlet wavelet transform power spectrum method confirms this relationship, but the estimation performance with spreading code lengths of 127 and 255 is similar at −6 dB. In contrast, the proposed method demonstrates perfect estimation accuracy irrespective of the spreading code length.

Figure 6 compares the chip rate estimation results using different spreading codes at various SNRs. In the context of DSSS systems, Gold sequences and m-sequences are commonly used as spreading codes owing to their excellent auto-correlation properties. These properties significantly minimize the interference between codes, making them strong candidates for robust signal estimation in noisy environments. The results in Figure 6 indicate that, when using the Morlet wavelet transform power spectrum method, the Gold sequence outperforms the m-sequence in terms of estimation performance. However, the proposed method demonstrates superior estimation accuracy for both the Gold sequence and the m-sequence, highlighting its versatility and effectiveness in real-world scenarios.

**Figure 7:**
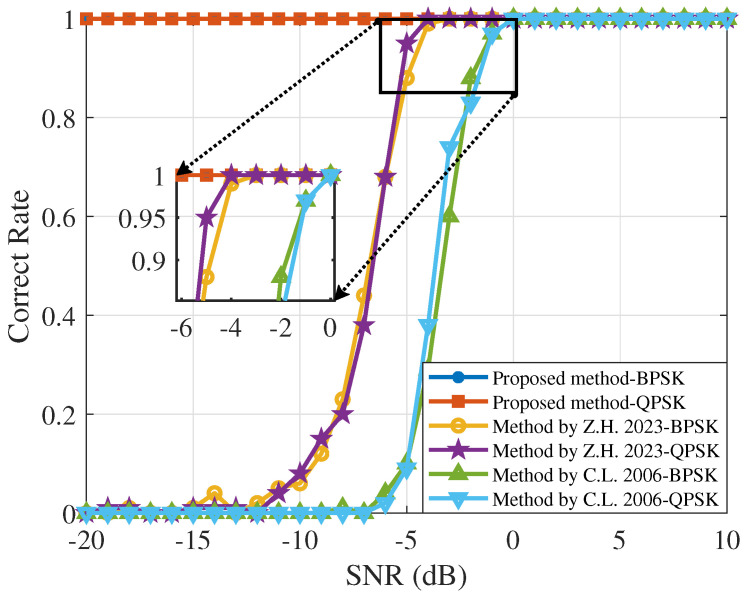
The performance curves of the Quadrature Phase Shift Keying (QPSK) and BPSK-modulated Direct Sequence Spread Spectrum (DSSS) signal chip rate estimation were compared under three different algorithms. Solid circles and squares represent the algorithm proposed in this paper, hollow circles and pentagrams represent the algorithm based on the Morlet wavelet transform power spectrum method [11], and upward-pointing and downward-pointing triangles represent the algorithm based on the Haar wavelet transform power spectrum method [10].

Figure 7 shows the performance of the estimation for the three algorithms applied to DSSS signals modulated with QPSK and BPSK at various SNRs. The results indicate that the Morlet wavelet transform power spectrum method generally outperforms the Haar wavelet transform power spectrum method. Specifically, for SNRs greater than −4 dB, the Morlet wavelet transform power spectrum method accurately estimates the chip rate of DSSS signals irrespective of the modulation scheme used. The Haar wavelet transform power spectrum method shows similar performance after the SNR reaches 0 dB. However, the performance difference between BPSK and QPSK is minimal at other SNR conditions. In contrast, the proposed method accurately estimates the chip rate of DSSS signals at low SNRs, regardless of the modulation used.

In summary, the simulation results show that our proposed method clearly outperforms traditional methods in anti-noise capability. Specifically, it accurately estimates the chip rate of DSSS signals in low SNR environments, independent of information code length, spreading sequence type, spreading code length, or modulation method. To further verify the robustness of the method, we then analyzed actual signals.

## 5. Acquisition and Analysis of Real Signals

In our experiments, an RTL-SDR device based on the RTL2832U+R820T2 chipset was used. The specific model used was the RTL2832U USB dongle, which is widely recognized in software-defined radio applications. This economical RTL-SDR device, which interfaces with a personal computer via USB 2.0 [16], was selected to capture the actual DSSS signal. Control and data acquisition were facilitated through simulation software. For the simulation experiments, an m-sequence with a spreading factor of 100 and a length of 127 was used to spread 10 data bits, which were then modulated using BPSK. The spreading rate and sampling rate were set to 1 MHz and 2 MHz, respectively. The relative error was used as the correct rate estimation, with an error of less than 0.01% considered an acceptable estimation.

**Figure 8:**
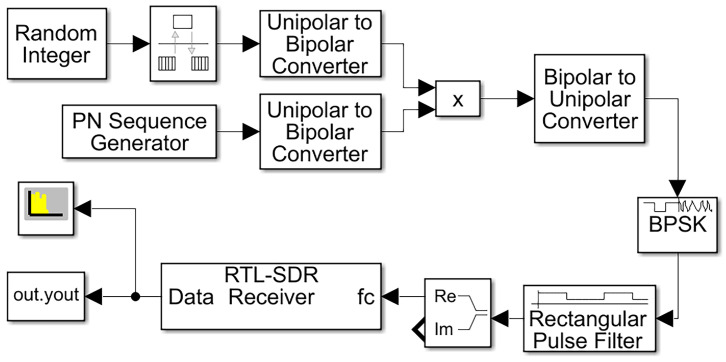
Simulink model for receiving a DSSS BPSK message.

Figure 8 depicts the block diagram of an RTL-SDR receiver for the acquisition of DSSS signals. The DSSS transmitter signal was constructed using modular components within simulation software. Data acquisition was conducted using the RTL-SDR receiver, and the output was saved in the ‘yout’ module.

**Figure 9:**
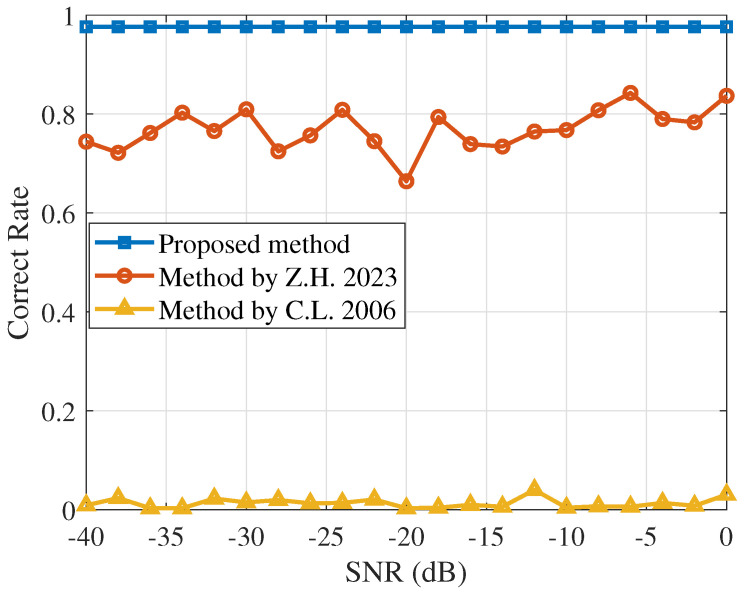
Simulation results for the measured data. The square line represents the algorithm proposed in this paper, the circle line represents the algorithm based on the Morlet wavelet transform power spectrum method [11], and the upper triangle line represents the algorithm based on the Haar wavelet transform power spectrum method [10].

Figure 9 shows that the proposed method consistently yields higher estimation rates compared to traditional methods when applied to actual data. Specifically, the results of the Haar wavelet transform power spectrum method align with theoretical analysis as it is sensitive to noise and thus struggles to accurately estimate the chip rate at low SNRs. The accuracy of the Morlet wavelet transform power spectrum method fluctuates due to varying wavelet scale selections across different SNRs in signal processing. On average, it achieves about 80% accuracy, outperforming the Haar wavelet transform power spectrum method. In contrast, the proposed method maintains a steadily high estimation accuracy of 97.6%.

## 6. Conclusions

This paper proposes the TF energy squeeze method for chip rate estimation. By performing post-processing operations on the wavelet transform, this method effectively focuses most of the ambiguous TF energy, enhancing signal characteristics. The optimization enhances the precision of the estimation of the chip rate of the direct-sequence spread spectrum signal, particularly in the context of high noise interference. To evaluate the method’s capability in processing both analog and real data signals, it was applied to real signals and quantitatively compared with other classical methods. The experimental results indicate that the method fully exploits the advantages of wavelet transform in TF post-processing, and exhibits high estimation performance in low SNRs environments. However, the method has some limitations. Reducing time operator error requires searching for the optimal position via N-order iteration, which increases computation complexity. Future work will focus on optimizing this aspect to reduce computational load and exploring the method’s application in various communication systems. 

## Figures and Tables

**Table 1:** Different wavelet basis simulation time(s).

Wavelet Basis Mode	Times
Mexican Hat wavelet	47.35 s
Meyer wavelet	45.36 s
Morlet wavelet	36.98 s

## Data Availability

This study did not report any data.
